# ‘It would help people to help me’: Acceptability of digital phenotyping among young people with visual impairment and their families

**DOI:** 10.1177/20552076231220804

**Published:** 2024-01-05

**Authors:** Bethany Higgins, Lee Jones, Kishan Devraj, Caroline Kilduff, Mariya Moosajee

**Affiliations:** 1 Institute of Ophthalmology, 4919 University College London, London, UK; 2 Division of Optometry and Vision Sciences, City, University of London, London, UK; 3 Research Directorate, BRAVO VICTOR, London, UK; 4 Moorfields Eye Hospital NHS Foundation Trust, London, UK; 5 376570 The Francis Crick Institute, London, UK

**Keywords:** Digital phenotyping, eHealth, smartphone application, visual impairment, acceptability

## Abstract

**Objectives:**

To explore the acceptability of an eHealth App for vision-related monitoring and symptom reporting among young people with a visual impairment and their parents.

**Methods:**

Qualitative investigation using virtual semi-structured focus groups (via Zoom software) of seven young participants with a genetic eye disorder including inherited retinal disease and structural eye abnormalities (e.g. microphthalmia), and 7 parents; all recruited from ocular genetic clinics at Moorfields Eye Hospital. Audio transcripts were analysed using thematic analysis.

**Results:**

Data were coded into six key themes: (1) increased involvement in care, (2) opportunity for less hospital-centric care, (3) better representation of visual impairment in a real-world setting, (4) trust in a reputable service provider, (5) harnessing data for health purposes and (6) intended purpose of the app. Both young people and their families were accepting of an eHealth app and felt they would be empowered by greater involvement in their care plan, if privacy of the data was retained, and information was managed correctly. While parents endorsed the opportunity for mental health tracking, young people were hesitant towards its inclusion.

**Conclusion:**

In summary, there was overall acceptability of an eHealth app among young people with a visual impairment and their parents. These findings will help to maximise the effective integration of digital phenotyping when monitoring and supporting young people experiencing sight loss.

## Introduction

Visual impairment in children and young people has a significant impact on psychological, educational and socioeconomic experience during childhood development and beyond into adulthood.^1^ Sight loss associated with genetic eye disorders such as inherited retinal diseases or structural eye abnormalities (e.g. microphthalmia) is rare in the general populace, but is estimated to represent a third of blind or severely visually impaired registrations in young children in the United Kingdom (UK).^2^ These cohorts are complex and heterogeneous, with a variety of different symptoms (e.g. nyctalopia and loss of peripheral or central vision). For most genetic eye diseases there is no established genotype–phenotype relationship, meaning clinical management may differ significantly from person to person.

Evidence suggests young people have a tendency towards disengaging from health services due to factors such as children rarely being involved in health-related decision-making^3^ and a paternalistic culture.^4^ Consequently, this age group may have an elevated risk of experiencing avoidable health problems.^5,6^ Disengagement is an important issue in paediatric ophthalmology as patient monitoring is essential to manage the impact of morbidities during key post-natal ocular development and visual maturation. To address these concerns, clinicians in the UK National Health Service (NHS) are increasingly searching for alternative, person-centred options for healthcare management that improve engagement and care plan compliance. Furthermore, UK government policy and investment are driving digitisation of the NHS,^7^ whereby technology is utilised to facilitate better communication between healthcare professionals and to enable the public to access the care they need efficiently. Therefore, the role of eHealth and digital communication in healthcare has grown significantly, enabling trusts to improve the quality of care and effectiveness of services.

eHealth is the empowerment and facilitation of health in individuals, families, and communities by means of digital information management.^8^ eHealth is not limited to telecommunication between patients and healthcare professionals, but can also comprise smartphone-based software used to generate clinically relevant data using elegant and creative methods, such as digital phenotyping. Digital phenotyping is the use of smartphone data or ‘digital footprint’ to study individual behaviour and physical well-being, first described by Torous et al.^9^ Data collected by a smartphone (e.g. location, keystrokes, and sleep metrics) may be correlated with clinical measurements, and these associations may help to identify disease modifications, such as progression. For instance, reduced outdoor activity in the dark, longer time required to type messages, or more prone to spelling errors while typing could all serve as indicators of clinically relevant symptomatic behaviour in people with visual impairment. The nature of digital phenotyping and the vast amount of data collected creates an opportunity for analysis via machine learning techniques which could potentially improve the diagnostic and prognostic validity of current clinical measures.

In the UK, 98% of Generation Z (people aged 16–24 years) use a smartphone.^10^ As young people are prolific users of smartphones, they are particularly well-suited to digital phenotyping. There is increasing interest in utilising application-based technology in smartphones to capture real-time symptoms and behaviours to meet clinical needs, particularly related to mental illnesses,^11^ and indeed there are smartphone apps designed to assess vision directly.^12,13^ However, digital phenotyping has not been explored in vision-related health and this includes both the impact of vision loss and specific behaviours and health-related parameters among individuals with vision loss. It has been reported young people feel they would benefit from access to digital communication with their health team^14^; however, the effectiveness of digital communication on improving health outcomes is equivocal.^15,16^ Parent–healthcare professional relationships are of paramount importance in young people's healthcare delivery, and it is unknown how the introduction of an eHealth app-based product would impact these relationships.^17^ Yet, eHealth tools have the potential to improve engagement and encourage greater self-management. In addition, eHealth has the capacity to reduce healthcare inequalities by engaging individuals who are considered underserved populations.^15^

Implementing digital phenotyping could help to build a picture of how individuals are affected by visual impairment in their daily lives, with the scope to modernise healthcare delivery by measuring outcomes which are important and relevant to patients themselves. This concept has not previously been explored in people with visual impairment. However, the research team are working towards the development of a smartphone app for the purpose of digital phenotyping in ophthalmology. Yet, before an application is developed it is of paramount importance to understand both children’s and parent's attitudes towards using app-based eHealth tools to aid in healthcare delivery. Thus, the aim of this study was to explore young patients’ and parents’ attitudes towards an eHealth App using digital phenotyping as an approach to monitor vision-related health.

## Methods

### Recruitment

Individuals meeting the study criteria (i.e. between the ages of 12 and 18 years with a genetically confirmed visual impairment) and their parents were invited to participate in a virtual, semi-structured focus group. Participants were recruited from Moorfields Eye Hospital (MEH) NHS Foundation Trust, London, UK. Purposive sampling was used whereby participants who met the inclusion criteria were invited to participate by their care provider (MM). Seven young people (and seven parents) were approached by a researcher previously unknown to them (BH) via email or telephone and agreed to participate. While seven people were unable to participate due to other commitments. See Table 1 for participant demographics.

**Table 1.** Participant demographics and clinical details for young people

ID	Age at the time of the focus group	Gender	Clinical diagnosis (causative gene if known)
C1	16	Female	Microphthalmia
C2	17	Male	Type 2 Usher syndrome (*USH2A*)
C3	15	Female	Early onset retinal dystrophy (*CRB1*)
C4	13	Male	Type 2 Usher syndrome (*USH2A*)
C5	14	Male	Type 2 Usher syndrome (*USH2A*)
C6	18	Male	Choroideremia (*CHM*)
C7	16	Female	Stargardt disease (*ABCA4*)

### Procedure

**Figure 1.**
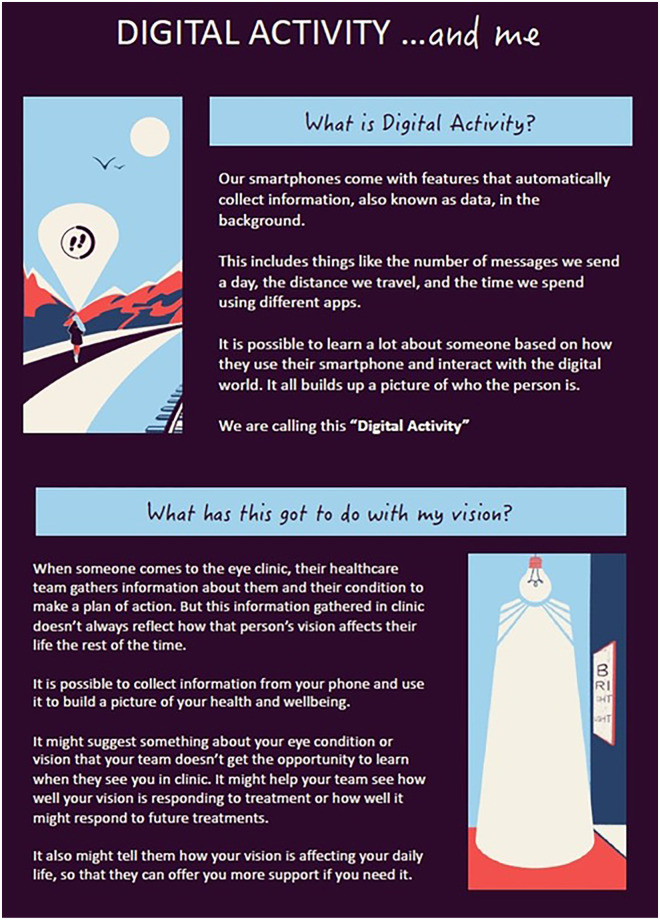
Exemplar section of the digital activity leaflet given to focus group participants.

Prior to the focus groups, an information leaflet was sent to participants and their parents to inform them about digital phenotyping (relabelled ‘digital activity’ for ease of understanding). The information leaflet was carefully designed for a young audience (see Figure 1). The purpose of the leaflet was to provide all participants with a basic understanding of digital phenotyping, describe the nature of information collected by an eHealth App (steps count and light levels) and reassure participants about the information that would remain private (content of messages and photos). All participants gave written informed consent before participating in this study.

A topic guide was devised by clinicians specialising in inherited retinal diseases and vision-related health (MM and CK), experts in qualitative analysis techniques (BH and LJ) and experts in eHealth and smartphone app development (KD) (see Supplemental Materials 1). Items included in the topic guide were based on an initial canvassing exercise whereby people with visual impairment were asked about their opinions on the concept of a digital phenotyping smartphone app. As the focus of the study was to assess acceptability among young people, a greater proportion of the topic guide focused on the patients alone, while a smaller session just with the parents was conducted at the end. In total, three virtual focus group sessions were organised. The first session involved the participation of five young individuals and five parents, while the subsequent two sessions were conducted separately, each comprising one young participant and one parent. While parents remained in the room while the patients participated in the focus group, they did not actively participate until the end. The young people were not present when the parents participated in the last part of the session. Focus groups were audio recorded and conducted virtually by a female Research Fellow with a background in psychology, a PhD in vision science and trained in qualitative research (BH). BH did not have any contact with the patients or parents prior to the focus group taking place. At the beginning of the focus group, the interviewer (BH) introduced themselves and the research team present (MM, LJ, and KD) and explained their role as facilitators in the session and the reasons for undertaking the research.

### Data analysis

Thematic analysis within a realist framework was used to analyse the data^18^ and a semantic approach was used to process the focus group transcripts to develop the themes. Focus group data were coded via NVivo V.11 (QSR International, Cambridge, Massachusetts, USA). Data were analysed by BH and interpretation of the coding was completed by both BH and LJ, then finalised with the entire research team. Codes were not predefined prior to analysis; hence an inductive approach was used to develop codes from the data. The research team recognised that data saturation had occurred when no new themes were found in the later focus groups. The Consolidated Criteria for Reporting Qualitative Research reporting guidance was followed (see Supplemental Materials 2). No datasets were generated or analysed during the current study.

## Results

Seven young people were recruited (*n*  =  3 female) aged between 13 and 18 years (median age 16 years) (see Table 1). All participants had a formal diagnosis of an inherited retinal disease (*n*  =  3 Usher syndrome, *n*  =  1 *CRB1*-related retinitis pigmentosa, *n*  =  1 choroideremia, *n*  =  1 Stargardt disease) or a structural eye abnormality (*n*  =  1 microphthalmia). Seven parents were also recruited (*n*  =  6 female) with no visual impairment and their relationship with the young people can be found in Table 2. The focus groups lasted between 1 h and 2 h.

**Table 2.** Participant demographics for parents.

ID	Gender	Caregiver of	Relationship to child
P1	Female	C1	Mother
P2	Female	C2	Mother
P3	Female	C3	Mother
P4	Female	C4 & C5	Mother
P5	Female	C6	Mother
P6	Female	C7	Mother
P7	Male	*	Father

*Parent 7 (P7) is father to a child with an inherited retinal disease who was approached to join the focus group but declined.

**Figure 2.**
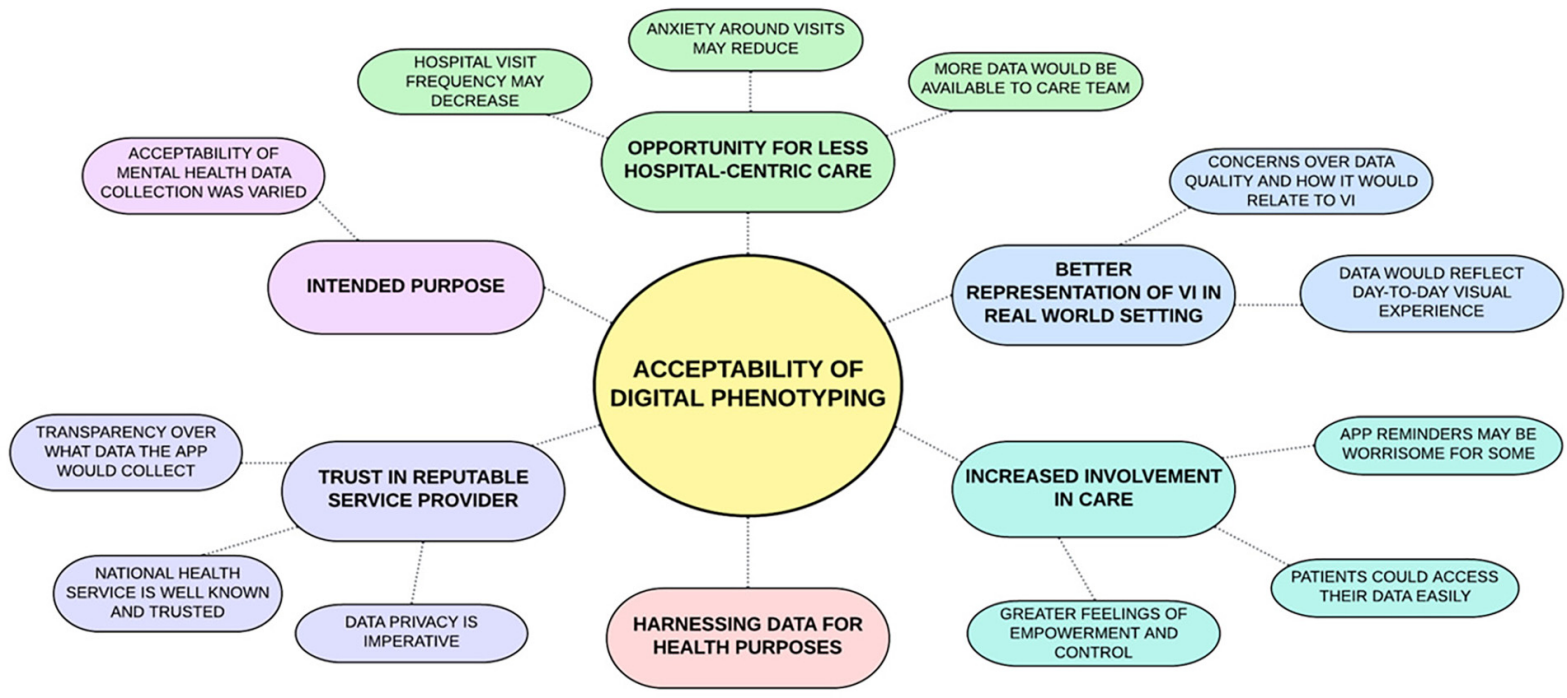
Diagram of key themes and sub-themes that emerged from focus group data.

Data were coded into six key themes relating to attitudes towards digital phenotyping using an eHealth app: (1) increased involvement in care, (2) opportunity for less hospital-centric care, (3) better representation of visual impairment in a real-world setting, (4) trust in reputable service provider, (5) harnessing data for health purposes and (6) intended purpose of the app. Direct quotations taken from the focus group transcripts are italicised and used to illustrate the chosen themes. All excerpts are annotated with the ID given to the corresponding participant. See Figure 2 for a diagram of the six themes and sub-themes.

### Increased involvement in care

A common theme across young participants' narratives was increased feelings of empowerment and control due to a sense of greater involvement in health management if an eHealth app was introduced to their care plan:

[An eHealth app would] reassure me that I’m doing something to try and keep track of it… I have some sense of control over it. (C2)

I think it could be quite empowering. (C3)

One young person felt an increased participatory role in their healthcare management would assist their care team in providing better support:

I would be willing to do it…it would help people to help me. (C4)

Another young person reflected that an eHealth app may assist with coming to terms with an unexpected sight impairment diagnosis:

It will help them come to grips with the fact that they have this condition, because they see it won’t change their life too much. (C6)

A priority area for both the young participants and the parents was to have greater access to their clinical data to help improve their understanding of the condition:

It's so hard to keep track of your vision, I think. Because the change is so slow and it's just quite a hard thing to describe anyway. So, any way to qualitatively describe it a bit better, I definitely think would be really useful. (C2)

One parent explained that having access to the smartphone-generated data could help to characterise their child's current visual status and thus help alleviate worry or anxiety:

He's bumping into this and he's doing this and doing that, and then all of the data that you’ve got is saying, well, actually, it's fine, then I’ll know it's me. (P5)

However, it was made apparent by young participants that the extent to which the app was acceptable was associated with the frequency of reminders (e.g. self-report symptom scales) to use the app, and that any such reminders would need to be tempered or personalisable, as consistent reminders of sight loss may be worrisome for some people:

I think if it was too often, it might get a bit depressing, maybe? Because like, you’re always just reminded, if it comes up on your phone like every day. (C3)

I feel like it could be helpful in the right circumstances, but in other circumstances, it’d be a bit oppressive. (C5)

I think the option to choose how often and the time would be really good, to be honest. Because I think it will really depend on the person how often they want to do it, and whether notifications annoy them or help them. (C2)

Parents reflected on the importance of reassuring users about the purpose of the eHealth app, highlighting that tracking declining visual function could be a cause for anxiety, particularly in cases where treatments were not yet available to be administered and monitored:

I’m very happy for there to be tracking…If you can say you’re doing this for a reason, and it is going to have a positive effect knowing that you’re declining at a particular time… Because it's ultimately quite a depressing thing to be tracking decline in vision. (P3)

It's a very depressing thing to watch it get worse but then I suppose that if there are treatments available, then hopefully you’d see an upward curve afterwards or you’d see a slowdown? (P4)

Seeing that trajectory and seeing that road ahead of maybe guide dogs and canes and seeing what that progression might look like. When you’re not there yet, and you’ve just got on the road, you’re just starting that journey, that in itself was quite fearful going to clinic. (P2)

### Opportunity for less hospital-centric care

A second theme was eagerness for care outside of the hospital setting. Anxiety and fear around hospital visits were reported by the parents, on behalf of their children and themselves:

I think it's a really great idea that maybe this is taking the fear out [of] seeing this annual check as being so fearful and maybe desensitising it a little. (P2)

I don’t know whether this app could help with firstly picking up things that she's not saying but even making the visit to the hospital less traumatic. (P6)

The hospital's not – we just don’t like going there. From that aspect, it stops a bit of upset if you don’t have to go as much. (P6)

One parent highlighted that an eHealth app would be particularly important to track outcomes of future potential treatments, with the scope to reduce the need to come into the clinic:

If there was a treatment that come out, [the eHealth app] would be very important…to see what the outcome of the treatment is, what's happening, and how it's working without again having to come to the hospital. (P6)

The young participants reported relief over the possibility of not having to visit the hospital as frequently and feeling they would be helping by not taking up appointments:

The fact that I don’t have to go in to see a doctor every three months or whatever to get a check-up, and they can just do it on the phone without the hassle of an appointment. It made me feel like I was being like really helpful. (C7)

This sentiment of possibly reducing the pressure on ophthalmology services was echoed by parents:

If there's no need for somebody to be on a yearly recall, you’re not clogging up your clinics. So it's monitoring everybody generally and actually calling in people when and as the need is, then you can get more people in, see more people more quickly. (P6)

Young participants also indicated that they feel they don’t get all the information they want from their hospital appointments and feel an eHealth app would offer them more information, outside the hospital setting:

I don’t really enjoy going to the appointments and check-ups because at the appointments, I don’t really get told a lot… if they could do that without me having to go in and just do it over the phone, it’d be much easier. (C7)

You only go to the hospital to get checked a few times a year or less and because sometimes changes can be so slow, it's hard to keep track of your sight and any changes that might be happening to it. So, any way to do that would definitely be useful. (C2)

Parents reported they’d feel happier and relieved with more information on a more regular basis at their disposal from an eHealth app:

Knowing, I think, is better than not knowing. So, it's that kind of feel of anxiety that's getting replaced very, very quickly with a feeling of relief, because you’ve been enlightened in terms of what has happened over a given period. (P7)

An app that maybe drip, drip, drip [information] is probably better… than an annual thing. (P2)

### Better representation of visual impairment in a real-world setting

Both parents and young people believed if the app was successful in accurately representing the everyday impact of visual impairment, it would give clinicians a better idea of the day-to-day experience of living with a visual impairment, and a more holistic view of visual function outside of the clinic setting:

If you’re checking on their mobility, their confidence levels, their health and wellbeing, their actual clinical output, then you’re coming from every kind of spectrum, then you can give them a full treatment. (P6)

Yet, there were some concerns regarding the quality of the data and how the app would relate to visual function:

The one major concern that I have would be the accuracy of it. It needs like a proof of accuracy, before. That would be my big thing. (C6)

I’m not really sure how all this stuff links to vision? I don’t know if it would always be a good indicator. Just because it's so broad and there are so many other things. (C3)

I don’t think it’d be accurate, because it's not always about you. It could be it's just raining one day. So, that's why you don’t go out. Or it could be hotter, so, you go out more with your friends. (C5)

### Trust in reputable service provider

A key factor affecting acceptability was due to participants having a relationship and high degree of trust with the organisation associated with the app. There was an emphasis that equivalent levels of trust may not be granted if the app was associated with companies outside of their care setting:

I have a huge amount of trust in Moorfields Eye Hospital. They’ve been outstanding….if it's a big, widespread thing, I might have a few more concerns. (P5)

I would trust that…the data was stored safely and securely. So, I wouldn’t have any concerns. (P5)

We totally trust the information is absolutely in the right hands and there's clearly nothing malign going on. (P2)

I trust the care team to have my data. (C7)

However, the need for **privacy** was also emphasised:

Where I go, the gradient of the light that I’m in, that kind of information is fine, but none of the more personal stuff. (C6)

There’d be concerns you’d be discriminated against, or things like that based on the data that's collected. (C2)

I definitely see the value in writing down how you feel and how things are going with your vision, but I’m not sure if I’d want all of that to be accessible. (C2)

Yeah, as long as it was obviously confidential. Just as long as it was just the care team seeing it. That's fine. (C7)

In an effort to retain privacy, it was made clear that **transparency** regarding exactly what the eHealth app would collect and why, would be required:

I think anyone is going to be uncomfortable to some extent, with that data being collected, but it's just got to be like presented in a way so everyone knows exactly what's being done with it and understands the value of it that it can provide to other people as well. (C2)

Particularly for parents who were concerned about geolocation data being shared with their children, clarity was considered imperative:

In terms of all the data types you proposed you’d be tracking, I would be, again, ensuring all the proper controls and checks are in place, I'm comfortable with all of that. Always a bit of a nervousness around things like location and things like that. (P7)

Obviously, all the necessary safeguards and checks and balances would need to be in place, which is standard. So, certainly, I’ll be entirely comfortable with [it]. (P7)

I think I’d probably need to have a lot of guarantees and reassurance of how that data was going to be managed and who was going to be using it. (P5)

### Harnessing data for health purposes

Participants acknowledged that their smartphones generate a significant amount of data contributing to their digital footprint. As such, it was gratifying for participants that the utility of using these data in a healthcare context was being investigated:

I’ve got a health thing that shows my steps, like my health trends and my distance travelled, that kind of thing. I already have that, so I would be completely fine if there was – if it was all in one app, which also helped with research for my eyes. I’d be more than on board with that. (C6)

I feel like the amount of times I press allow cookies when I was in primary school. I must be tracked by so many things. It doesn’t really matter anymore. (C5)

What's the point of not doing this if your data is already being used? They’re just using it in a better way? (C4)

I think like most apps and social media, and everything already do it. So, you might as well do it for something that could be beneficial. (C3)

### Intended purpose of app

There was some disagreement between participants regarding the acceptability of eHealth app depending on the intended use case. For example, young participants were happy for an eHealth app to support their vision-based health, but were less tolerant of an app being used to also track their mental health:

As long as it's just related to my vision, I don’t think I would mind it at all. But I definitely would feel a bit more – like it would feel a bit more invasive to talk about how I feel and more personal stuff like that. (C2)

I know that it's good, and it's a good thing to collect. But I don’t think I particularly enjoy writing it all out. (C7)

Personally, I just don’t feel it's necessarily…. It just feels a little invading to other stuff that is going on in life other than visual-based stuff. (C1)

In contrast, other young participants were not averse to the idea of mental health tracking, but wanted reassurance that any associations wouldn’t be assumed to be directly related to vision:

I’d say quite keen. I think it's good, I think. As long as you consider other things in life. Like other than just vision, I think definitely, yeah. If it's targeted right. (C3)

Parents were all unanimously supportive and encouraging of mental health tracking, believing it to be essential:

I have no concept, really, about it…which I think would be really useful. Because I struggle to really get from him how he feels. (P5)

I mean, with teenage boys, they don’t really talk much. (P2)

Would I like to know, or would my growing child like to know how their mental health changes with their change in sight? I think that's absolutely essential to know. (P7)

I do think there's a direct link between children's mental health and their visual impairment and their differences against their peers. So, I think it is a good idea to track it. (P1)

## Discussion

There is a drive for healthcare providers to make better use of data and digital technology to improve the quality and efficiency of services.^19^ Yet, in the field of vision sciences, progress may be hindered by a lack of knowledge of patient's attitudes towards app-based eHealth products. Children with complex and rare vision-related conditions represent a cohort in need of effective healthcare management, due to the heterogeneity of their conditions and care plans. We found that young people and their families are generally accepting of an eHealth app for the purposes of digital phenotyping. However, smartphone data privacy and management must be considered if this approach is to become a more regular component of healthcare.

Overall, participants were largely accepting and would engage with an eHealth app to monitor their vision-related health, believing this concept to be a good use of their digital data. However, evidence of a division was found between parents and young people on the desirability of mental health tracking via an eHealth app. While parents unanimously felt mental health tracking to be essential, young participants were unsure of its relevance to their vision-related healthcare management. Questions from participants arose during focus groups highlighting the need for transparency and clarity regarding what data are collected, its storage and a proof of concept study illustrating data quality and reliability. This information should be relayed to patients when introducing the concept of digital phenotyping and eHealth applications.

A key factor affecting willingness to use the app among young people was the opportunity to have a greater role in their healthcare, thereby providing a source of empowerment. International agreements such as the United Nations Convention on the Rights of the Child dictate that children have a legal right to express their views on and to be actively involved in their healthcare.^20^ However, children and families are rarely involved in health-related decision-making,^3^ which may play a subsequent role in young people's disillusionment and subsequent disengagement with their care plans. Our findings revealed a continued desire of participants to feel involved in monitoring and managing their visual impairment. While this result is associated specifically with vision-based healthcare, it is similar to results found by other studies into children's involvement in wider healthcare management.^21^

Keeping track of eye health was an area of difficulty for participants, and an eHealth app offering a simple and accessible way to gain insights about their vision outside of the clinic was appealing. Ensuring young people with sight loss understand their condition is integral for them to implement coping strategies to aid their adjustment to living with a visual disability.^22^ Therefore, a digital tool may help achieve greater health literacy and could potentially facilitate Instrumental Activities of Daily Living such as medication adherence and the effective use of technology, which in turn can lead to better health outcomes.^23^ Yet, it was also suggested that frequent reminders of sight loss may be detrimental to mental health. For example, frequent reminders of a steadily decreasing trajectory in vision may not be helpful, especially when no treatment is available.^24^ Previous research with glaucoma patients indicates frequent monitoring and self-reporting of symptoms can lead to maladaptive rumination, whereas they were previously unperturbed by their condition. As a result, unidentified negative feelings may impact a patient's self-efficacy and could potentially lead to patients developing low affect or depression.^25^ Some respondents, particularly parents, highlighted that if this eHealth app would be used in conjunction with a treatment in a clinical trial, they could see its benefit in delivering this information to young people. Otherwise, the eHealth app may become a troubling reminder. This is an important issue raised by this study and consideration of the way (and the why) visual impairment information is delivered to young people via an eHealth app is essential. To potentially mitigate this issue, eHealth apps could be developed as personable, in that the user has control over the frequency of reminders and can tailor it to their preference and amend it if it feels burdensome.

Participants reported some fears associated with their eye care appointments. Vision impairment is associated with greater symptoms of anxiety and depression, particularly in inherited retinal eye disease^26–28^ and uncertainty regarding clinical visits may contribute towards the psychological sequela of vision impairment. While mitigating feelings of anxiety *during* clinic visits is needed, interventions like an eHealth app may begin to reduce concerns surrounding these visits by providing patients with information associated with their eye and vision health and thus removing the ‘fear of the unknown’.

An eHealth app may enable effective patient prioritisation by estimating the level of need for each patient with a recommended follow-up period. Such an approach could make available valuable clinical services that can be redirected to individuals in greater need of clinical review. Opportunity for patient prioritisation through the app was recognised by both young people and parents and reflects public concern that the NHS is over-burdened.^29^ Ophthalmology departments are particularly strained, for example, 7.4 million ophthalmology clinical visits took place between 2021 and 2022, the highest out of all treatment specialities.^30^ Creative solutions are required to help relieve the pressure, and digital phenotyping was considered by participants to have a promising role in improving patient management.

Overall acceptability of the eHealth app was based upon the respondent's innate trust in data management and protection of the NHS and their clinical care team. A recent poll of the UK general public reported nurses and doctors to be among the most trustworthy professions.^31^ This is a positive result, identifying that both young people and their parents believe this passive digital data would be respected and stored safely by an NHS Trust and that eHealth would be a welcomed direction for care plan management. This is despite the recent fall in public satisfaction in the NHS.^32^ However, the use of eHealth apps raises some ethical issues, like the need for privacy of the collected smartphone data. This concern was repeatedly brought up during focus groups. Particularly for parents considering location data of their children. This is a common concern associated with eHealth research^33^ and in the UK, the app would adhere to regulatory body guidelines,^34^ thus ensuring data protection.

Participants recognised that smartphone-based data was automatically being collected by their device and an eHealth app would potentially repurpose this data for visual health-related use. Digital phenotyping data can be collected with little interaction of the user and removes bias associated with self-reporting.^35^ The participants were also agreeable to interacting with an app (e.g. self-report questionnaires), but some also expressed concern over data quality, in that they weren’t sure *how* the data would relate to their vision. The feasibility of smartphone-based digital phenotyping has been assessed in physical diseases such as asthma, amyotrophic lateral sclerosis, and spinal disease^36–38^ as well as psychological disease.^11^ Data collected was found to be clinically relevant and comparable to standardised assessments and self-reports in both paediatric^36^ and adult cohorts.^37,38^ However, to the author's knowledge, there have been no studies that have assessed if digital phenotyping of smartphone data can be used in the field of visual health. This is the natural next step of this overall study by our research team and will form our future work.^39^ However, in the first instance, the acceptability of the concept of digital phenotyping in a visually impaired cohort must be determined first.

In some cases, there was an aversion to the concept of mental health tracking through an app. For example, there was a perceived irrelevance of mental health to eye health from young people. Evidence has found patients are less likely to disclose psychological problems with their healthcare provider due to fear of the consequences of the consultation, such as unwanted involvement of mental health services.^40^ Furthermore, concerns regarding mental health among children have been found to increase into adolescence.^41,42^ Meanwhile, parents in this study were enthusiastic at the prospect of an app to help monitor the mental health of their children. It was raised particularly by those parents with teenagers that they are unsure how their child feels in general and towards their visual impairment. Not only did parents indicate they felt mental health was closely aligned with their children's visual impairment, but also that an eHealth app would alleviate their concerns. This disparity between the two groups is significant as help-seeking for mental health services is typically driven by the parent rather than the child, who is then faced with several challenges that may prevent service engagement (e.g. parental problem recognition, service accessibility, and financial barriers).^43^ The negative impact of poor mental health in childhood extends into adulthood and can lead to a reduction in life satisfaction^44^ and an increased likelihood of mental health problem development.^45^ Therefore, this finding represents an important consideration when developing an eHealth app that aims to explore mental health alongside another health component.

This study opens avenues for further research in the area of digital phenotyping in visually impaired cohorts. For example, while digital phenotyping has been explored in young people in other health domains, namely in the field of psychiatry,^9,11,33^ this is the first study to the author's knowledge exploring attitudes towards digital phenotyping via a smartphone in a cohort with visual impairments. This study incorporated the views of young people and their families and addresses the need to include these stakeholders in health-related decision-making. The information garnered from this study will inform the development of an eHealth app specifically designed for use in a visually impaired cohort that will be subject to a feasibility assessment. This evaluation should further assess factors that will influence the acceptability of the app, including cost, ease of use, and aesthetics. Such a tool may have a wide range of applications across the ophthalmology sector, such as in clinical trials to track changes in visual function or self-reported quality of life in the comfort of participants’ home environments.

We recognise some limitations to our study. We recruited from only one large hospital centre (MEH) and the cohort was relatively small (*n*  =  14; *n*  =  7 young people and *n*  =  7 parents), which may limit the generalisability of our findings to an extent. Yet, as we conducted our study using virtual means, our findings represent views from a group of young people with rare visual impairments who live across the UK. In addition, our study relied primarily on the perspectives of mothers in the focus group. We acknowledge that the acceptability of the app to healthcare teams and other stakeholders, such as fathers, caregivers, and healthcare professionals, plays a crucial role in its successful implementation and adoption. While the insights from mothers served as a valuable starting point for understanding user perspectives, future research should consider a more diverse range of perspectives to ensure a comprehensive understanding of the topic. A further limitation may be that as some focus groups were conducted as a group, participants may have been hesitant to answer questions candidly on sensitive topics such as mental health. Despite this, participants surveyed were outspoken about the importance of talking about mental health, but were hesitant in agreeing to mental health tracking. While this is an important consideration, where focus groups were conducted alone (for participants C6 and C7), the same sentiments on mental health tracking were expressed by the young people and their families.

## Conclusion

In summary, young people with visual impairments and their parents were largely accepting of digital phenotyping via a smartphone app to be potentially incorporated into their health care plan. To ensure effective implementation, the need for privacy, transparency regarding the data collected and a proof of concept study illustrating data quality and reliability are necessary. While parents unanimously felt mental health tracking to be essential, young participants were unsure of its relevance to their vision-related healthcare management and a balance would need to be found in tracking vision-related mental health. These findings will be used to aid the development of an eHealth smartphone app to enable testing of digital phenotyping in a visually impaired cohort.

## Supplemental Material

sj-docx-1-dhj-10.1177_20552076231220804 - Supplemental material for ‘It would help people to help me’: Acceptability of digital phenotyping among young people with visual impairment and their families Click here for additional data file. Supplemental material, sj-docx-1-dhj-10.1177_20552076231220804 for ‘It would help people to help me’: Acceptability of digital phenotyping among young people with visual impairment and their families by Bethany Higgins, Lee Jones, Kishan Devraj, Caroline Kilduff and Mariya Moosajee in DIGITAL HEALTH

sj-docx-2-dhj-10.1177_20552076231220804 - Supplemental material for ‘It would help people to help me’: Acceptability of digital phenotyping among young people with visual impairment and their families Click here for additional data file. Supplemental material, sj-docx-2-dhj-10.1177_20552076231220804 for ‘It would help people to help me’: Acceptability of digital phenotyping among young people with visual impairment and their families by Bethany Higgins, Lee Jones, Kishan Devraj, Caroline Kilduff and Mariya Moosajee in DIGITAL HEALTH
