# Supplementary material for: ‘It would help people to help me’: Acceptability of digital phenotyping among young people with visual impairment and their families
Source: Digit Health. 2024 Jan 5;10:20552076231220804. doi: 10.1177/20552076231220804 (PMC10771050; doi:10.1177/20552076231220804)
Supplement: sj-docx-1-dhj-10.1177_20552076231220804 - Supplemental material for ‘It would help people to help me’: Acceptability of digital phenotyping among young people with visual impairment and their families [file sj-docx-1-dhj-10.1177_20552076231220804.docx]

**TOPIC GUIDE FOR FOCUS GROUP WITH YOUNG PEOPLE**

**PROJECT:** eHealth application for young people with visual impairments: eHelp or eHindrance?

**
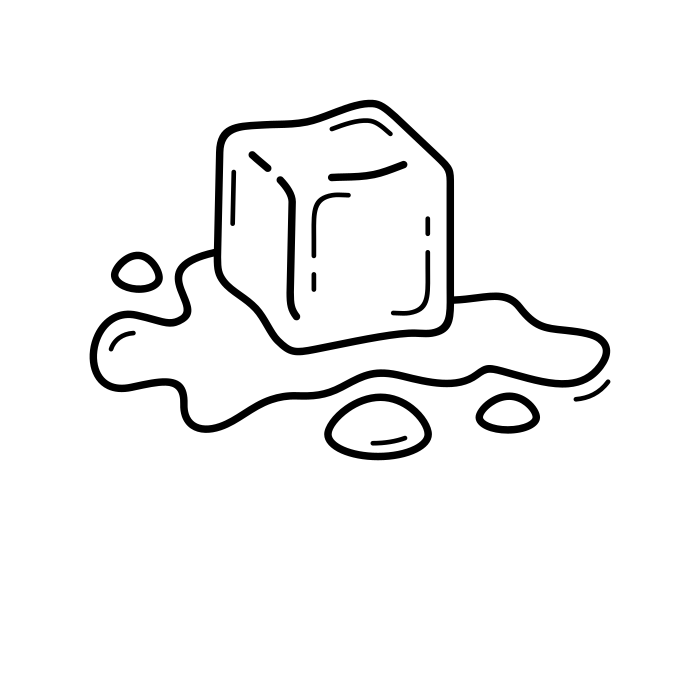
SECTION 1: ICEBREAKERS**

**"There are no wrong answers in this group. We are interested in finding out how you use your smartphone. And by that we mean a mobile phone with other functions e.g. messaging, apps, social media and internet access. Examples of these include iPhones, and Androids"**

- How many hours do you spend time using your phone?
  - *[P] 12 hours - so all day?*
  - *[P] between 9 and 12 hours?*
  - *[P] between 6 and 9 hours?*
  - *[P] between 3 and 6 hours?*
  - *[P] between 1 and 3h?*
  - *[P] less than 1h?*
- What apps do you use most and why?
  - *[P] Messaging apps, Video apps, Music apps*
- Do you use apps related to health and wellbeing and why?
  - *[P] Step counters; sleep recorded, wellbeing, mindfulness*
- Do you use any special software to make your phone easier to use?
  - *[P] contrast, magnification, screen reader*
  - *[P] does this work well with the apps you use?*

**
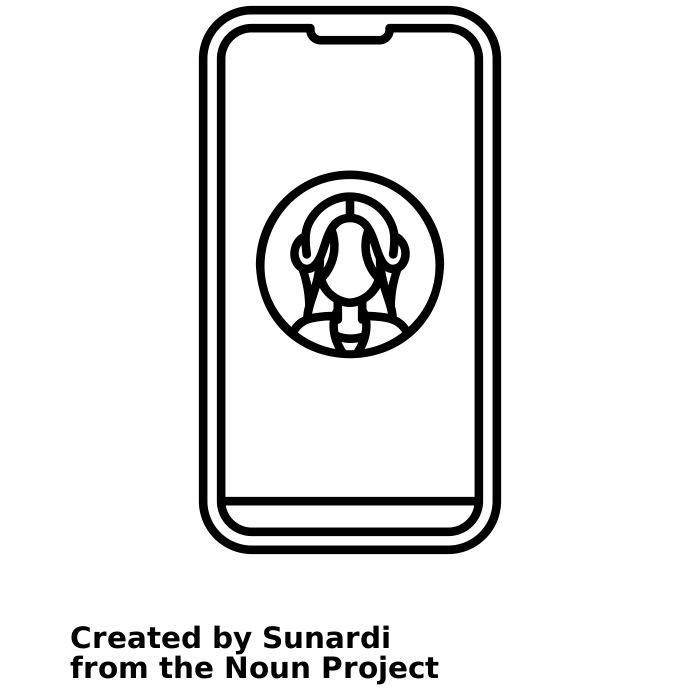
SECTION 2: SMARTPHONES AND DIGITAL ACTIVITY**

**A smartphone captures a lot of different types of information about the user. We can learn a lot about someone based on how they use their phone e.g. the number of messages we send a day, the distance we travel, and it all builds up a picture of who the person is and what they are like.**

**For example, if your inbuilt step counter on your phone registers that you walk 15,000 steps a day, it is suggesting that you are someone who can walk and who is quite fit.**

**It is possible to collect this information from your phone and use it to build a picture of your health and wellbeing.**

**It's important to note that when we talk about collecting this data from your smartphone, we are not able to see what you say in your messages or what you're googling. Just that you have sent e.g three messages today, and you have been on the internet.**

- What are your first thoughts on digital activity?
  - *[P] What do you think the advantages might be for you?*
  - *[p] Do you think there would be disadvantages for you?*
- Would you find it acceptable if digital activity played a role in your health care plan?
  - *[P] Would you consider it if your health care team offered it?*
  - *[P] Do you have concerns about it? What are they?*

**So as we discussed, digital activity would not include data such as what you’ve been messaging and googling or any of your photos of videos. It would include data like: where you go, how frequently you call people, how long it takes you to type.**

- Are there some types of data you would feel more or less comfortable with sharing with your care team?
  - *[P] Map data (where you go), calls (frequency), texts (time taken) and why*

**There are two ways data is collected in digital activity: passive and active. In passive phenotyping, data that your phone already records is collected in the background (e.g. time spent using apps). In active phenotyping, data you actively record is collected (e.g. filling in a questionnaire about how you feel)**

- How would you feel about using an app to record your symptoms or answer questions related to your health?
  - *[P] Interested? Helpful? Time-consuming?*
- What kind of feedback would you want on this collected data?
  - *[P] e.g. step count, how well you sleep*
  - *[P] Frequency of feedback?*
  - *[P] Alerted to change?*
- How regularly do you think you would use an app to record your symptoms?
  - *[P] Do you think you would need reminders/notifications? what would make you want to use the app, what would make you stop using it?*
- How frequent would you want notifications from an app?
  - *[P] How many per week? Per month?*
  - *[P] How many is too many?*

**
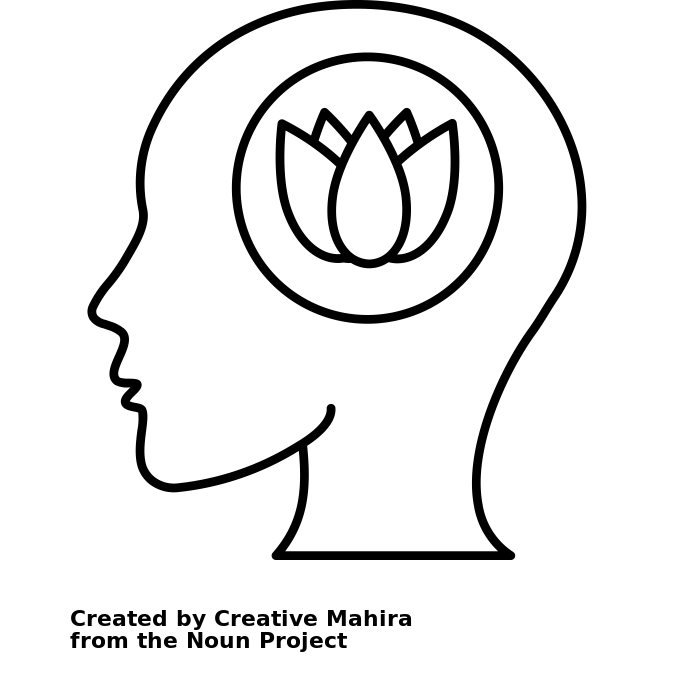
SECTION 3: DIGITAL VISION ACTIVITY AND MENTAL WELLBEING**

- How appropriate do you feel it is to monitor things like your mental wellbeing in relation to your sight impairment?
  - *[P] What do you like about the idea?*
  - *[P] What are your concerns about it?*
- How would it make you feel if you were asked to report your symptoms through an app?
  - *[P] Empowered? Distressed?*
- To what extent would having an app such as the one we've described impact on you in terms of how you think/feel about your VI?
  - *[P] Empowered? Distressed?*

**Any other comments for the team? Any questions from the team to the group?**


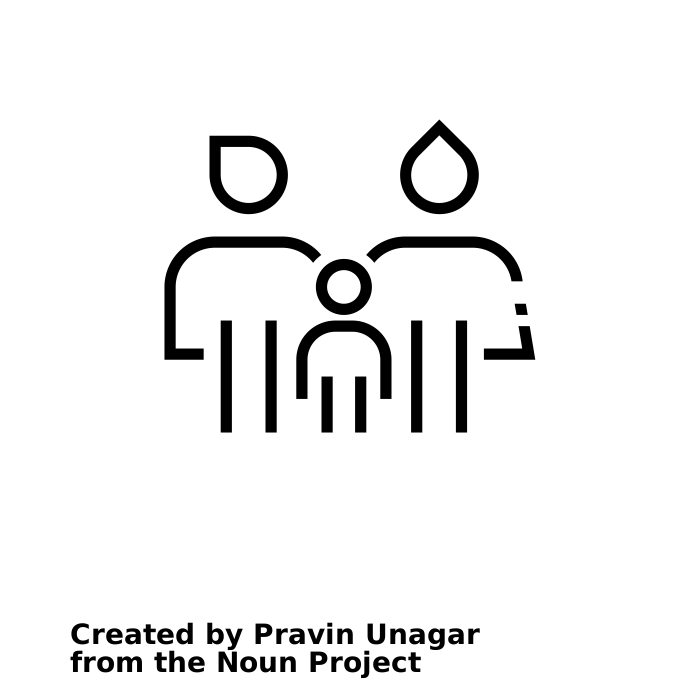
**SECTION 4: PARENT’S VIEWS**

- What are your thoughts on the use of digital activity in your child’s health care plan?
  - *[P] advantages, disadvantages*
- What role do you see digital activity playing in your child’s health care plan?
  - [*P] would you consider it if your health care team offered it to your child?*
  - *[P] Do you have concerns about it?*
- Are there some types of your child’s data you would not like to share with their care team?
  - *[P] map data (where they go), calls (frequency), texts (time taken)*
- How appropriate do you feel it is to monitor things like your child’s mental health in relation to their sight impairment?
  - *[P] Do you have concerns about it?*
